# Human Papillomavirus-Associated Subsequent Malignancies among Long-Term Survivors of Pediatric and Young Adult Cancers

**DOI:** 10.1371/journal.pone.0070349

**Published:** 2013-08-05

**Authors:** Rohit P. Ojha, Joseph E. Tota, Tabatha N. Offutt-Powell, James L. Klosky, Timothy D. Minniear, Bradford E. Jackson, James G. Gurney

**Affiliations:** 1 Department of Epidemiology and Cancer Control, St. Jude Children’s Research Hospital, Memphis, Tennessee, United States of America; 2 Department of Epidemiology, Biostatistics, and Occupational Health, McGill University, Montreal, Quebec, Canada; 3 Office of Infectious Disease Epidemiology, Division of Public Health, Delaware Health and Social Services, Dover, Delaware, United States of America; 4 Department of Psychology, St. Jude Children’s Research Hospital, Memphis, Tennessee, United States of America; 5 Department of Infectious Diseases, St. Jude Children’s Research Hospital, Memphis, Tennessee, United States of America; 6 Department of Preventive Medicine, University of Alabama at Birmingham, Birmingham, Alabama, United States of America; 7 Department of Epidemiology, Biostatistics, and Environmental Health, University of Memphis School of Public Health, Memphis, Tennessee, United States of America; The Catalan Institute of Oncology (ICO), Spain

## Abstract

Long-term survivors of pediatric and young adult (PAYA) cancers have a high incidence of subsequent neoplasms, but few risk factors other than cancer treatment have been identified. We aimed to describe the burden of human papillomavirus (HPV)-associated malignancies among survivors of PAYA cancers to assess whether HPV infections might be a reasonable area of future etiologic research on subsequent malignancies in this population. We used longitudinal data from 9 population-based registries of the Surveillance, Epidemiology, and End Results program collected between 1973 and 2010 to assemble a cohort of individuals who were diagnosed with any cancer between the ages of 0 and 29 years and survived at least 5 years post-diagnosis. We estimated sex-specific standardized incidence ratios (SIRs) with corresponding 95% confidence limits (CL) of HPV-associated subsequent malignancies (cervical, vaginal, vulvar, penile, anal, tongue, tonsillar, and oropharyngeal). Our study population comprised 64,547 long-term survivors of PAYA cancers diagnosed between 1973 and 2010. Compared with females in the general US population, female PAYA cancer survivors had a 40% relative excess of HPV-associated malignancies overall (SIR = 1.4, 95% CL: 1.2, 1.8). Compared with males in the general US population, male PAYA cancer survivors had a 150% relative excess of HPV-associated malignancies overall (SIR = 2.5, 95% CL: 1.9, 3.4). Our findings suggest an excess of HPV-associated malignancies among PAYA cancer survivors compared with the general US population. We hypothesize that a portion of subsequent malignancies among PAYA cancer survivors may be directly attributable to HPV infection. This hypothesis warrants exploration in future studies.

## Introduction

Long-term survivors of pediatric and young adult (PAYA) cancers have an excess incidence of subsequent malignancies in various organ systems relative to what would be expected in the general population [1]–[8]. Subsequent malignancies among PAYA cancer survivors are primarily related to curative treatment, particularly radiation [9]. Nevertheless, treatment alone does not account for all subsequent malignancies. Genetic predisposition has been the main alternate hypothesis for explaining excess subsequent malignancies, but several genetic association studies among survivors of PAYA cancers suggest that common polymorphisms have little influence on the incidence of subsequent malignancies [10]–[19]. Consequently, hypothesis-generating studies may be useful for identifying additional factors to explore in relation to subsequent malignancies among PAYA cancer survivors.

Persistent infection with oncogenic human papillomavirus (HPV) types, particularly HPV-16 and -18, is a well-established cause of cervical cancer [20]. The development of vaginal, vulvar, penile, anal, and oropharyngeal cancers is also partially attributable to oncogenic HPV types [21]–[25]. The proportion of PAYA cancer survivors who engage in high-risk sexual behaviors that increase the risk of HPV infection is comparable to the general population [26], [27], which suggests that HPV infection could be a risk factor for subsequent malignancies among PAYA cancer survivors. One approach for generating evidence about whether HPV infection may be a risk factor for subsequent malignancies among PAYA cancer survivors is to describe the burden of HPV-associated malignancies (i.e. malignancies for which HPV infection is an etiologic factor) in this population, which is currently unknown. Therefore, we aimed to describe the burden of HPV-associated malignancies among long-term survivors of PAYA cancers by estimating the cumulative incidence of such malignancies and comparing the incidence relative to the general United States (US) population.

## Methods

### Study Population

We used longitudinal data from 9 population-based registries of the Surveillance, Epidemiology, and End Results (SEER) program [28] collected between 1973 and 2010 to assemble a cohort of individuals who were diagnosed with any cancer between the ages of 0 and 29 years and survived at least 5 years post-diagnosis. Although data from 13 SEER registries are available to analyze subsequent malignancies, these data only cover the period between 1992 and 2010, which truncates the duration of follow-up. A shorter follow-up could result in an underestimate of the burden of HPV-associated malignancies because of insufficient duration for the natural course of disease. The 9 SEER registries include Atlanta, Connecticut, Detroit, Hawaii, Iowa, New Mexico, San Francisco-Oakland, Seattle-Puget Sound, and Utah [29]. These registries comprise ∼10% of the US population with nearly 100% case ascertainment in the coverage area using active case-finding methods [29]. The SEER program is well-known for its representativeness of the US population and data completeness, comparability, accuracy, and timeliness [29]. Case data include baseline demographics, primary cancer site, first-course therapy (e.g. type of radiation therapy), and follow-up information on subsequent malignancies and vital status [28]. Our maximum age for young adult survivors (up to age 29 years when diagnosed with first malignancy) was based on the definition of young adults according to a recent SEER monograph on adolescent and young adult cancers [30].

### Ethics Statement

The publicly available de-identified SEER data used for this analysis are exempt from institutional review board approval (Code of Federal Regulation 46.101 b(4) [31]).

### Outcomes

Our outcomes of interest were restricted to subsequent malignancies for which current evidence suggests an etiologic relation with HPV (i.e. HPV-associated malignancies): cervical, vaginal, vulvar, penile, anal, oropharyngeal, tongue, and tonsillar cancers [21]–[25]. These outcomes were identified in the SEER data using the variable “Site recode B ICD-O-3/WHO 2008,” which classifies cancers according to location and International Classification of Diseases of Oncology version 3 (ICD-O-3) and is updated with the World Health Organization (WHO) Classification or Tumors of Haematopoietic and Lymphoid Tissues [32]. In addition to these individual outcomes, we aggregated outcomes based on general anatomy to yield categories for HPV-associated anogenital malignancies (cervical, vaginal, vulvar, penile, and anal) and HPV-associated head and neck malignancies (oropharyngeal, tongue, and tonsillar). PAYA survivors contributed person-time to the cohort from diagnosis of the first cancer until incidence of HPV-associated malignancy, death, time until lost to follow-up, or the end of the study period (December 31, 2010), whichever occurred first.

### Data Analysis

We used person-time and outcome data for PAYA cancer survivors to estimate the cumulative incidence of all HPV-associated subsequent malignancies based on Fine and Gray’s proportional subdistribution hazard model to account for the competing risk of death [33]. Person-time and outcome data for PAYA cancer survivors (i.e. observed number of HPV-associated malignancies) and corresponding data for the general US population standardized by age, race, and calendar-year (i.e. expected number of HPV-associated malignancies) were used to estimate sex-specific standardized incidence ratios (SIRs) with an assumed Poisson distribution for site-specific HPV-associated malignancies and for the two aggregated subgroups. We estimated 95% Wald confidence limits (CL), a large sample method [34] ^p.240–243^, for the SIR when the expected number of cases was ≥5 and mid-*P*-value CL, a small sample method [34] ^p.253–254^, when the expected number of cases was <5.

### Sensitivity Analysis

PAYA cancer survivors may interact with the healthcare system more often than the general US population (e.g. 87% of 5-year survivors of pediatric cancer reported general medical contact [35]), which could alter disease detection rates between the two groups and manifest as differential outcome misclassification. We explored the potential impact of outcome misclassification for PAYA cancer survivors and the general US population in a sensitivity analysis using a range of values for sensitivity (Se) and false-positive rate (Fr; the number of false-positive diagnoses per person-year) for HPV-associated malignancies overall. The adjusted counts of observed and expected cases of HPV-associated malignancies overall were computed using the following formulae [34] ^eq.19–13^:
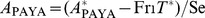


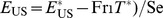
where A* and E* are the original unadjusted numbers, and T* is person-years. Given the SEER program’s extensive efforts to confirm diagnoses of submitted cases [29], Fr is expected to be near 0 (i.e. no false-positive diagnoses would be expected). Therefore, the above formulae reduce to *A*_PAYA_ = A*_PAYA_/Se and *E*_US_ = E*_US_/Se [34] ^p.359^, and the SIRs adjusted for misclassification were computed as *A*_PAYA_/*E*_US_.

## Results

Our study population comprised 64,547 long-term survivors of PAYA cancers diagnosed between 1973 and 2010 in the US. *Table 1* summarizes the characteristics of our study population. Briefly, the majority of PAYA cancer survivors were female (53%), and White survivors comprised the largest racial subgroup (84%). For primary malignancies that occurred among females and males, the most common was Hodgkin lymphoma (12%). The cumulative incidence of all HPV-associated subsequent malignancies was 0.71% (95% CL: 0.50%, 0.97%), with a median duration of follow-up of 17 years (interquartile range [IQR] = 10–25 years). The median age at diagnosis of a subsequent malignancy commonly associated with HPV infection was 38 years (IQR = 32–46).

**Table 1:** Characteristics of pediatric and young adult (PAYA) cancer survivors diagnosed between 1973 and 2010 in the United States.

Characteristic	
*Age at diagnosis; n (%)*	
0–9 years	12,000 (19)
10–19 years	13,245 (21)
20–29 years	39,302 (61)
*Female; n (%)*	34,299 (53)
*Race; n (%)*	
White	54,385 (84)
Black	5,117 (7.9)
Other	5,045 (7.8)
*Primary cancer diagnosis; n (%)*	
Brain or central nervous system	5,889 (9.1)
Breast^a^	1,959 (5.7)
Cervical^a^	2,619 (7.6)
Head and neck	1,437 (2.2)
Hodgkin lymphoma	7,838 (12)
Non-Hodgkin lymphoma	3,472 (5.4)
Leukemia	6,676 (10)
Melanoma (cutaneous)	6,778 (11)
Ovarian^a^	1,670 (4.9)
Sarcoma	1,721 (2.8)
Testicular^b^	6,523 (22)
Thyroid	6,888 (11)
Other	11,077 (17)
*Radiation; n (%)*	19,780 (31)
*Years of follow-up; median (IQR* c *)*	17 (10–25)
*Cumulative incidence of HPV-associated subsequent malignancies (95% confidence limits)*	0.71% (0.50%, 0.97%)
*Age at diagnosis of HPV-associated subsequent malignancy; median (IQR)*	38 (32–46)

a Among females;

b Among males;

c Interquartile range.

*Table 2* summarizes the overall, site-specific, and radiation-specific SIRs of HPV-associated malignancies for female PAYA cancer survivors. Compared with females in the general US population, female PAYA cancer survivors had a 40% relative excess of all HPV-associated malignancies (SIR = 1.4, 95% CL: 1.2, 1.8), largely attributable subsequent head and neck cancers (overall SIR = 3.3, 95% CL: 2.2, 5.2). We observed a relative excess of all site-specific HPV-associated malignancies except cervical cancer (SIR  = 1.0, 95% CL: 0.77, 1.3) and oropharyngeal cancer (no observed cases) among female PAYA cancer survivors. A relative excess of all site-specific malignancies persisted for female PAYA cancer survivors regardless of first-course radiation status except for cervical cancer among females not treated with radiation (SIR = 0.88, 95% CL: 0.63, 1.2), tonsillar cancer among females not treated with radiation (SIR = 0.88, 95% CL: 0.04, 4.3), and oropharyngeal cancer (no observed cases). For HPV-associated anogenital malignancies (overall SIR = 1.3, 95% CL: 1.0, 1.6), the highest relative excess for female PAYA cancer survivors was observed for vaginal cancer (SIR = 6.1, 95% CL: 3.0, 11). For HPV-associated head and neck malignancies, the highest relative excess for female PAYA cancer survivors was observed for tongue cancer (SIR  = 3.8, 95% CL: 2.2, 6.0).

**Table 2:** Standardized incidence ratios (SIRs) for human papillomavirus (HPV)-associated malignancies among female survivors of pediatric and young adult (PAYA) cancers, 1973 to 2010.

					95% CL^b^	
Cancer type		Observed	Expected	SIR	LL^c^	UL^d^
*HPV-associated cancers* a	Overall^e^	105	72.64	1.4	1.2	1.8
	No radiation^f^	64	53.16	1.2	0.93	1.5
	Radiation^g^	41	19.48	2.1	1.6	2.9
*Anogenital cancers*	Overall	85	66.64	1.3	1.0	1.6
	No radiation	52	48.63	1.1	0.81	1.4
	Radiation	33	18.01	1.8	1.3	2.5
Anal^h^	Overall	7	4.36	1.6	0.70	3.2
	No radiation	5	3.31	1.5	0.55	3.3
	Radiation	2	1.05	1.9	0.32	6.3
Cervical	Overall	55	55.05	1.0	0.77	1.3
	No radiation	35	39.90	0.88	0.63	1.2
	Radiation	20	15.15	1.3	0.85	2.0
Vaginal	Overall	9	1.48	6.1	3.0	11
	No radiation	5	1.12	4.5	1.6	9.9
	Radiation	4	0.36	11	3.5	27
Vulvar	Overall	14	5.75	2.4	1.4	4.1
	No radiation	7	4.30	1.6	0.71	3.2
	Radiation	7	1.45	4.8	2.1	9.5
*Head and neck*	Overall	20	6.00	3.3	2.2	5.2
	No radiation	12	4.53	2.6	1.4	4.5
	Radiation	8	1.47	5.4	2.5	10
Tongue	Overall	16	4.25	3.8	2.2	6.0
	No radiation	11	3.18	3.5	1.8	6.0
	Radiation	5	1.07	4.7	1.7	10
Tonsillar	Overall	4	1.49	2.7	0.85	6.5
	No radiation	1	1.14	0.88	0.04	4.3
	Radiation	3	0.35	8.6	2.2	23
Oropharyngeal	Overall	0	0.26	0	0	Undefined
	No radiation	0	0.21	0	0	Undefined
	Radiation	0	0.05	0	0	Undefined

a Includes anal, cervical, vaginal, vulvar, tongue, tonsillar, and oropharyngeal cancers;

b CL =  Confidence Limit;

c LL =  Lower Limit;

d UL =  Upper Limit;

e *n* = 34,299 female cancer survivors;

f *n* = 24,310 female cancer survivors not treated with radiation;

g *n* = 9,989 female cancer survivors treated with radiation;

h Includes anus, anal canal, and anorectum.

*Table 3* summarizes the overall, site-specific, and radiation-specific SIRs of HPV-associated malignancies for male PAYA cancer survivors. Compared with males in the general US population, male PAYA cancer survivors had a 150% relative excess of all HPV-associated malignancies (SIR = 2.5, 95% CL: 1.9, 3.4). We observed a relative excess of all site-specific HPV-associated malignancies among male PAYA cancer survivors except for oropharyngeal cancer (no observed cases). The relative excess persisted regardless of first-course radiation status except for penile cancer (no observed cases among males PAYA cancer survivors without first-course radiation therapy). For HPV-associated anogenital malignancies (overall SIR = 3.2, 95% CL: 1.7, 5.4), the highest relative excess for male PAYA cancer survivors was observed for penile cancer (SIR = 4.1, 95% CL: 1.0, 11). For HPV-associated head and neck malignancies (overall SIR = 2.3, 95% CL: 1.7, 3.3), the highest relative excess for male PAYA cancer survivors was observed for tongue cancer (SIR = 2.8, 95% CL: 1.9, 4.3).

**Table 3:** Standardized incidence ratios (SIRs) for human papillomavirus (HPV)-associated malignancies among male survivors of pediatric and young adult (PAYA) cancers, 1973 to 2010.

					95% CL^b^	
Cancer type		Observed	Expected	SIR	LL^c^	UL^d^
*HPV-associated cancers* a	Overall^e^	45	17.95	2.5	1.9	3.4
	No radiation^f^	30	11.95	2.5	1.8	3.6
	Radiation^g^	15	6.00	2.5	1.5	4.1
*Anogenital cancers*	Overall	12	3.81	3.2	1.7	5.4
	No radiation	7	2.53	2.8	1.2	5.5
	Radiation	5	1.28	3.9	1.4	8.7
Anal^h^	Overall	9	3.08	2.9	1.4	5.4
	No radiation	7	2.04	3.4	1.5	6.8
	Radiation	2	1.04	1.9	0.32	6.4
Penile	Overall	3	0.73	4.1	1.0	11
	No radiation	0	0.49	0	0	Undefined
	Radiation	3	0.24	13	3.2	34
*Head and neck*	Overall	33	14.14	2.3	1.7	3.3
	No radiation	23	9.42	2.4	1.6	3.7
	Radiation	10	4.72	2.1	1.1	3.8
Tongue	Overall	22	7.73	2.8	1.9	4.3
	No radiation	17	5.14	3.3	2.1	5.3
	Radiation	5	2.59	1.9	0.71	4.3
Tonsillar	Overall	11	5.72	1.9	1.1	3.5
	No radiation	6	3.82	1.6	0.64	3.3
	Radiation	5	1.9	2.6	0.96	5.8
Oropharyngeal	Overall	0	0.69	0	0	Undefined
	No radiation	0	0.46	0	0	Undefined
	Radiation	0	0.23	0	0	Undefined

a Includes anal, penile, tongue, tonsillar, and oropharyngeal cancers;

b CL =  Confidence Limit;

c LL =  Lower Limit.

d UL =  Upper Limit;

e *n* = 30,248 male cancer survivors;

f *n* = 20,457 male cancer survivors not treated with radiation;

g *n* = 9,791 male cancer survivors treated with radiation;

h Includes anus, anal canal, and anorectum.

*Table 4* summarizes the results of our sensitivity analysis which explored the potential impact of outcome misclassification on our SIR estimates for HPV-associated malignancies. A difference ≥30% in sensitivity of cancer detection between female PAYA cancer survivors and females in the general US population would nullify or reverse the observed relative excess of HPV-associated malignancies overall. In contrast, our results suggest that the relative excess of HPV-associated malignancies overall would persist even with a 30% difference in sensitivity of cancer detection between male PAYA cancer survivors and males in the general US population.

**Table 4:** Assessment of sensitivity to potential outcome misclassification when estimating the relative excess of HPV-associated malignancies among pediatric and young adult (PAYA) cancer survivors compared with the general United States (US) population.

Scenario	PAYA cases		General US population		SIR^a^
	Sensitivity	False-positive rate^b^	Sensitivity	False-positive rate	
*Females*					
1	1.0	0	1.0	0	1.4
2	1.0	0	0.90	0	1.3
3	1.0	0	0.80	0	1.2
4	1.0	0	0.70	0	1.0
5	1.0	0	0.60	0	0.87
6	1.0	0	0.50	0	0.72
7	0.90	0	0.90	0	1.4
8	0.90	0	0.80	0	1.3
9	0.90	0	0.70	0	1.1
10	0.90	0	0.60	0	0.96
11	0.90	0	0.50	0	0.80
12	0.80	0	0.80	0	1.4
13	0.80	0	0.70	0	1.3
14	0.80	0	0.60	0	1.1
15	0.80	0	0.50	0	0.90
*Males*					
1	1.0	0	1.0	0	2.5
2	1.0	0	0.90	0	2.3
3	1.0	0	0.80	0	2.0
4	1.0	0	0.70	0	1.8
5	1.0	0	0.60	0	1.5
6	1.0	0	0.50	0	1.3
7	0.90	0	0.90	0	2.5
8	0.90	0	0.80	0	2.2
9	0.90	0	0.70	0	1.9
10	0.90	0	0.60	0	1.7
11	0.90	0	0.50	0	1.4
12	0.80	0	0.80	0	2.5
13	0.80	0	0.70	0	2.2
14	0.80	0	0.60	0	1.9
15	0.80	0	0.50	0	1.6

a Standardized incidence ratio after applying classification rates for PAYA cases and the general US population;

b Assumed to be negligible because of an extensive confirmation process for cases submitted to the SEER program.

## Discussion

Our hypothesis-generating study aimed to describe the burden of HPV-associated malignancies among long-term PAYA cancer survivors. The cumulative incidence of all subsequent HPV-associated malignancies among PAYA cancer survivors was 0.71%, which suggests a modest absolute burden of disease. Our results also suggest that female and male PAYA cancer survivors have a relative excess of most HPV-associated malignancies compared with the general US population. In particular, a relative excess of head and neck malignancies compared with the general population is consistent between male and female PAYA cancer survivors. Furthermore, the relative excess of most site-specific HPV-associated malignancies persists regardless of first-course radiation status among female and male PAYA cancer survivors.

Despite cervical cancer representing approximately half of all subsequent HPV-associated malignancies among female PAYA cancer survivors, our results suggest that cervical cancer incidence is similar between female PAYA cancer survivors and the general population. One plausible explanation is that cervical cancer is screen-detectable and cervical cancer screening rates are similar between female PAYA cancer survivors and females in the general US population [36], which may prevent progression to invasive disease equally between the groups. An alternate explanation is that the duration of follow-up was insufficient for cervical cancer incidence among younger female PAYA cancer survivors. For example, the median duration of follow-up in our study was 17 years, which equates to a 9 year old female being age 26 years at the end of follow-up. This age of follow-up is considerably younger than the mean age of cervical cancer incidence (48 years [37]). Continued follow-up of this cohort may provide further insight.

Additional sources of uncertainty should be considered when interpreting our results. One *a priori* consideration included potential outcome misclassification because of differential rates of cancer detection between PAYA cancer survivors and the general population, which was explored in a sensitivity analysis. The results of our sensitivity analysis suggest that the difference in sensitivity of cancer detection would have to be at least 30% lower among females in the general US population than among female PAYA cancer survivors to nullify the relative excess of HPV-associated malignancies overall. The difference required to nullify the relative excess would have to be even more extreme than 30% between male PAYA cancer survivors and males in the general US population. We speculate that such large differences in the sensitivity of detecting HPV-associated malignancies between PAYA cancer survivors and the general US population are unlikely given that screening is available for cervical cancer and the symptoms associated with the other HPV-associated malignancies are likely to prompt medical attention. Nonetheless, the actual detection rates of HPV-associated malignancies among PAYA cancer survivors and the general US population are unknown. Consequently, the results of our sensitivity analysis offer simulated quantitative evidence that the relative excess of HPV-associated malignancies overall observed in our study is unlikely to be fully explained by outcome misclassification, particularly for male PAYA cancer survivors.

The SEER data used in our analysis include information about radiation as first-course therapy, but information is unavailable about the use of radiation to treat a recurrent cancer [38], [39]. This issue is more relevant to the stratified estimates for PAYA cancer survivors not treated with radiation. In particular, PAYA cancer survivors treated with radiation for cancer recurrence, but not for first-course therapy, could have a misclassified radiation therapy status. If classification errors in therapeutic radiation exposure are related to the true values of HPV-associated malignancies among these survivors, such differential misclassification could result in an overestimate of the corresponding SIR. Nonetheless, cancer recurrence is the most common cause of death among pediatric cancer survivors, and often occurs between 5 and 9 years post-diagnosis [40], which could preclude the development of an HPV-associated malignancy. Consequently, we speculate that our estimates may not be overly sensitive to bias from differential misclassification of radiation status.

The SEER program’s high case ascertainment rates for initial cancers within population-based coverage areas reduce the potential for underestimating the expected number of cases in the general US population when estimating an SIR [29]. An often-cited concern is that the complete ascertainment of subsequent cancers among cancer survivors is limited to individuals who remain in the region covered by the SEER registries where the initial cancer was diagnosed [38], [39]. Individuals who emigrate from the SEER region and have a subsequent cancer may thus be misclassified as not having a subsequent cancer, an issue that may be relevant given our young and mobile study population. Recent empirical evidence suggests that bias from emigration may not be a serious concern when using SEER data for pediatric cancer survivors [41]. Even if it were a concern, misclassification of subsequent cancer status because of emigration would downward-bias our SIR estimates of HPV-associated subsequent cancers among PAYA cancer survivors (i.e. if complete information on subsequent malignancies were available, the observed number of cases and thus the SIR could actually increase).

In summary, our findings suggest an excess of HPV-associated malignancies among PAYA cancer survivors. This relative excess is not fully attributable to radiation therapy, particularly among males. The relative excess of subsequent malignancies such as those of the head and neck could be partly explained by factors such as smoking [42], but HPV infection has gradually replaced smoking as the major risk factor of concern for head and neck malignancies [43]. Furthermore, childhood cancer survivors report modestly lower rates of smoking than the general US population [44].

Given the available evidence, we hypothesize that a portion of subsequent malignancies among PAYA cancer survivors may be directly attributable to HPV infection. HPV infections, based on direct measurements among individuals, have been associated with subsequent malignancies among adult cancer survivors [45], but similar evidence based on measurement of HPV infection status is not available among PAYA cancer survivors. Our findings may thus be useful for stimulating research to explore the relation between HPV infections and subsequent malignancies among long-term survivors of PAYA cancers.
